# Has Extracorporeal Gas Exchange Performance Reached Its Peak?

**DOI:** 10.3390/membranes14030068

**Published:** 2024-03-17

**Authors:** Foivos Leonidas Mouzakis, Ali Kashefi, Flutura Hima, Khosrow Mottaghy, Jan Spillner

**Affiliations:** 1 Institute of Physiology, Medical Faculty, RWTH Aachen University, 52074 Aachen, Germany; 2 Department of Thoracic Surgery, Medical Faculty, RWTH Aachen University, 52074 Aachen, Germany

**Keywords:** gas exchange, partial pressure, hyperbaric, atmospheric pressure, silicone, diffusion membrane

## Abstract

Extracorporeal gas exchange therapies evolved considerably within the first three–four decades of their appearance, and have since reached a mature stage, where minor alterations and discrete fine-tuning might offer some incremental improvement. A different approach is introduced here, making use of modern, purely diffusive membrane materials, and taking advantage of the elevated concentration gradient ensuing from gas pressure buildup in the gas chamber of the oxygenator. An assortment of silicone membrane gas exchangers were tested in vitro as per a modified protocol in pursuance of assessing their gas exchange efficiency under both regular and high-pressure aeration conditions. The findings point to a stark performance gain when pressurization of the gas compartment is involved; a 40% rise above atmospheric pressure elevates oxygen transfer rate (OTR) by nearly 30%. Carbon dioxide transfer rate (CTR) does not benefit as much from this principle, yet it retains a competitive edge when higher gas flow/blood flow ratios are employed. Moreover, implementation of purely diffusive membranes warrants a bubble-free circulation. Further optimization of the introduced method ought to pave the way for in vivo animal trials, which in turn may potentially unveil new realms of gas exchange performance for therapies associated with extracorporeal circulation.

## 1. Introduction

The evolution of extracorporeal gas exchange has been monumental ever since its conceptualization by Hooke (1635–1703), and even more so after the first open-heart surgery was performed by Gibbon (1903–1973) in 1953 [1]. Particularly the devices responsible for the gas exchange itself, dubbed artificial lungs but most commonly known as oxygenators, experienced profound progress in the second half of the 20th century. From the maiden application of bubble oxygenators to film/screen implementations, and prior to the deposition of the “direct contact” approach by the first flat-sheet membrane oxygenators, several decades have gone by [2]. Membrane oxygenators sacrificed gas-exchange performance to some extent in favor of hemocompatibility, making them an instant hit in long-term therapies [3]. The invention of hollow fiber fabrication technology brought about the first semi-permeable microporous capillary membranes, which outshined older oxygenator variants by scoring big in every aspect [4,5].

Polypropylene (PP) and polymethylpentene (PMP) hollow fiber mats have since monopolized the oxygenator scene, establishing the fundamental design principles of the contemporary gas exchanger. Regardless of the fiber bundle shape (stacked or wound), the average adult oxygenator requires approx. 1.8–2.0 m^2^ of capillary membrane to deliver adequate performance up to 7 L min^−1^ (e.g., Medos Hilite 7000, Xenios AG, Heilbronn, Germany, Quadrox PLS, Maquet GmbH, Rastatt, Germany). Barring an increase in surface area (*A_m_*) and/or any advancements in material science offering enhanced mass transfer coefficients (*k_c_*), packing density is the only variable available at the moment that may improve the performance of a hollow fiber membrane oxygenator (HFMO). By shifting towards more densely packed bundles, hydraulic diameter decreases just as blood’s boundary layer does, thus reducing gas diffusion resistance [6]. Gas exchange performance and priming volume naturally profit from this arrangement; regrettably a configuration detrimental to blood pressure drop, shear stress and blood trauma. In pursuance of avoiding acute side-effects and balancing out the impact of each and every one of the aforementioned parameters, a 50% packing density is usually preferred for conventional extracorporeal circulation applications.

**Figure 1:**
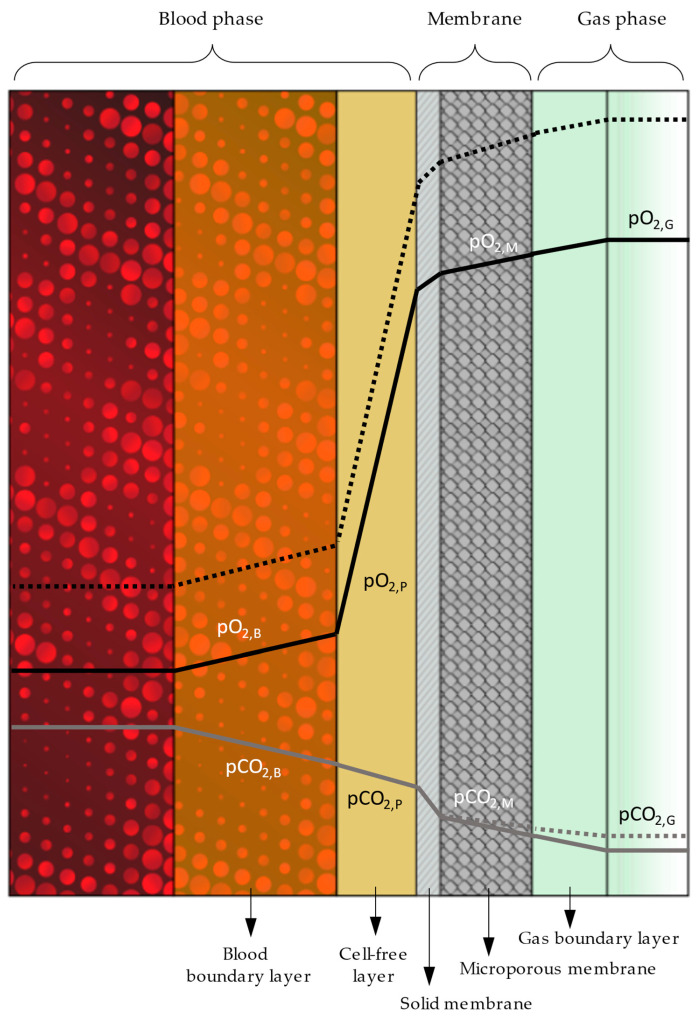
Graphic illustration of the diverse layers spanning between blood and gas phases during extracorporeal membrane gas exchange. Simultaneously shown is a qualitative representation of the resistance to gas transfer in terms of oxygen (*pO*_2_) and carbon dioxide partial pressure (*pCO*_2_). Continuous and dashed lines denote normobaric and hyperbaric gas delivery, respectively.

In order to better comprehend the kinetics of gas exchange in HFMOs and how the individual parameters influence performance, it is necessary to delve deeper into gas diffusion, and to investigate the resistance that the diverse layers and boundaries across the gas pathway pose. Figure 1 illustrates the intricate stratification stretching between the gas and blood phases in any microporous membrane oxygenator. The rightmost side represents gas flow and the corresponding boundary layer (directly adjacent, depicted in a slightly darker tone). Immediately after comes the microporous membrane that might feature a solid envelope to prevent plasma leakage (cross-hatched layer). Further to the left, on the outside surface of this skin, extends the cell-free marginal layer consisting solely of plasma [7]. Finally, between the cell-free layer and the erythrocyte-rich layer at the leftmost side lies the boundary layer of blood, which also contains red blood cells (RBCs), albeit in lower concentrations [8].

As the depicted course (continuous line) of oxygen partial pressure *pO*_2_ across the distinct strata denotes (Figure 1), the resistance to gas diffusion is nearly negligible for each transition, with the exception of the solid membrane and the cell-free marginal layer that markedly impede gas transfer. Considering the futility of attenuating resistance by altering the membrane’s packing density, reinforcing the potential of partial pressure seems the only alternative to improve gas diffusion. This option, however, is also limited once oxygen fraction, *FiO*_2_, reaches 1.0. In a novel approach, the concentration of oxygen is artificially increased by employing a mechanism that constricts the oxygenator’s gas outlet, leading to pressure buildup in the oxygenator’s gas compartment. An elevated positive pressure in the gas phase augments *pO*_2_ potential, prompting an upsurge in oxygen diffusion (dashed line).

Contrary to oxygen, carbon dioxide does not benefit from the concept of hyperbaric gas delivery. Following the opposite trend to oxygen molecules, carbon dioxide’s diffusion into the gas phase is in fact impeded by the hyperbaric barrier, resulting in impaired CO_2_ elimination rates. Implementation of higher gas flow/blood flow ratios alleviates this handicap to a great extent, just like physiological hyperventilation. Consolidation of these two techniques ought to accommodate for oxygen and carbon dioxide transfer rates (OTR and CTR) alike.

In an attempt to fathom the uncharted potential of hyperbaric gas delivery in extracorporeal gas exchange applications, an experimental study has been designed with one key parameter in mind: pushing an oxygenator past its typical limits of performance. In vitro investigations with diverse oxygenator modules, combined with normo-/hyperbaric gas supply conditions, put the concept’s feasibility to the test and permit a quantitative evaluation of the gas exchange efficacy attainable.

## 2. Materials and Methods

The migration and/or formation of gas bubbles in the bloodstream during any extracorporeal circulation therapies available nowadays must be avoided at all cost. Maintaining a bubble-free blood flow through the blood compartment of an oxygenator is of paramount importance when pressure in the gas chamber starts to build up. Hence, it is mainly purely diffusive solid membranes, or at least membranes featuring a solid skin, that may qualify for this purpose.

With the above limitations in mind, a solid-silicone fiber oxygenator of yesteryear (Mera Silox 0.8) was selected as a benchmark for testing silicone-coated microporous capillary fibers [9]. The concept of treating microporous capillary membranes with silicone to generate a purely diffusive solid envelope on their surface is by no means novel [10]. However, recent advancements in the field [11] brought forth a truly competitive—both in terms of performance and value—product, befitting hyperbaric gas supply regimens. The complete inventory of gas exchangers implemented in this study is summarized in Table 1, along with some essential technical specifications.

**Figure 2:**
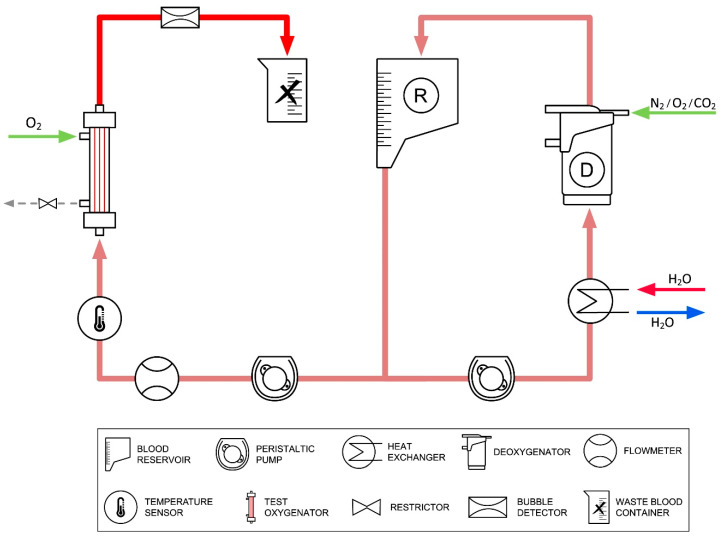
Graphical representation of the experimental setup. A flow control valve (restrictor) at the gas outlet permits pressure buildup in the gas compartment, thus facilitating hyperbaric gas supply.

In vitro investigations have been carried out with fully heparinized porcine blood to determine the influence of hyperbaric gas delivery on gas transfer efficacy. The extracorporeal circuit comprised two-roller/peristaltic pumps and a heater–cooler device (Stoeckert Instrumente GmbH, Munich, Germany), a deoxygenator (a commercial oxygenator assuming the role of the human body to dissipate oxygen and emit CO_2_), a filter, and a blood reservoir. Blood flow rate was monitored by an ultrasonic flowmeter (Transonic, Ithaca, NY, USA), whereas gas flowmeters (Platon, Vienna, Austria) were implemented for the regulation of gas supply. A bubble detector, integrated into the pump console, alerted to any gas bubbles in the bloodstream. Figure 2 displays said test setup.

Instead of following the standard (ISO 7199:2016) [12], the experimental protocol (see Table 2) was preemptively modified to accommodate a greater margin for oxygenation. In addition, higher gas flow/blood flow ratios were implemented (sweep gas *FiO*_2_ = 1.0) to counter tarnished CO_2_ removal under hyperbaric conditions. To gauge the actual gain of hyperbaric gas delivery, all the gas exchangers available were additionally tested under identical conditions in normobaric operation. Venous and arterial (i.e., pre- and post-oxygenator) blood samples, drawn at standard intervals, were analyzed using a blood gas analyzer (Radiometer, Copenhagen, Denmark) to obtain accurate readings, which are vital for the estimation of oxygen and carbon transfer rates (OTR–CTR).

## 3. Results

Each of the tested gas exchangers was subjected to the same experiment under identical testing conditions to assess its average performance at least three times. The standard deviation between all iterations was negligible (<1%) and is not therefore depicted.

**Figure 3:**
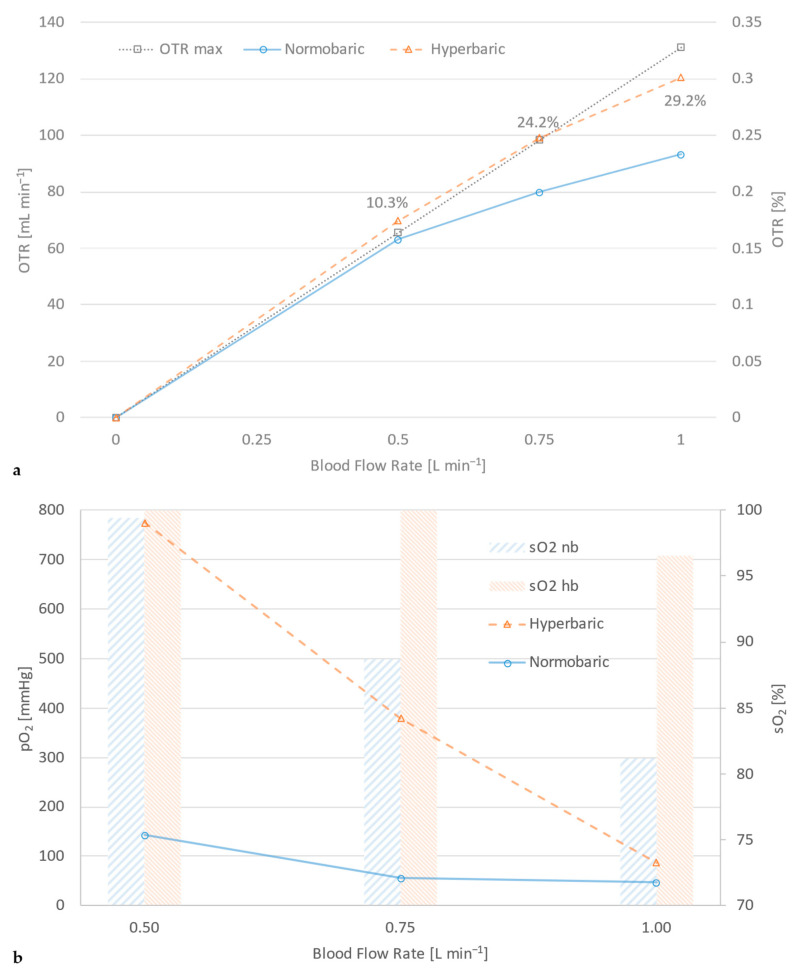
Gas exchange performance of the same oxygenator at hyperbaric and normobaric operation in terms of (**a**) oxygen transfer rate, (**b**) oxygen saturation and partial pressure.

Testing any machine or device to assess its performance customarily requires some sort of reference or benchmark to compete against. Often, in gas exchange investigations, another commercial product is employed as a means of gauging efficiency. Likewise, an arbitrary target value/parameter may be established as a reference with respect to pathophysiological indications. *OTR max* is defined as the gas transfer equivalent to fully saturated blood (*sO*_2_ = 100%), with an arterial partial pressure of oxygen, *pO*_2_, of 200 mmHg. Oxygen transfer rate under normobaric conditions does not fare well against this standard, as Figure 3a reveals. Application of hyperbaric gas delivery raises the efficacy of the same module to such heights (nearly 30% above normobaric operation) that it might actually surpass that of the *OTR max* levels. In order to provide a solid grasp of the attainable performance gain, Figure 3b offers a behind-the-scenes glance of the action by accentuating the difference in oxygen saturation and partial pressure between normo- and hyperbaric operation.

**Figure 4:**
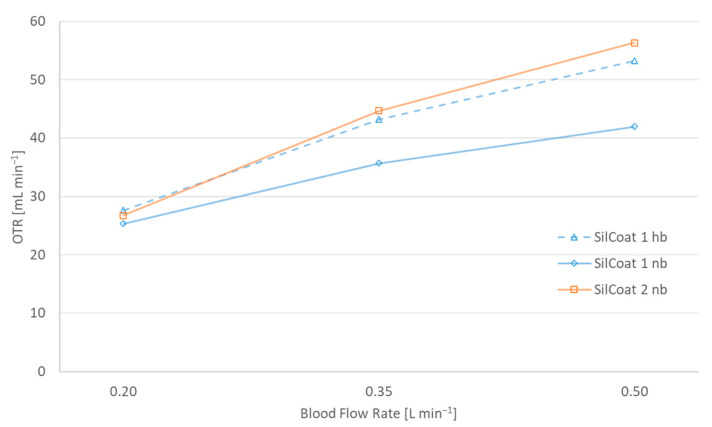
Comparison of oxygen transfer rate between a 0.7 m^2^ gas exchanger at normo- and hyperbaric operation and another one with 1.4 m^2^ surface area at normobaric conditions.

The improved gas exchange capacity of an oxygenator at hyperbaric operation can be compared to increasing the effective surface area of its membrane without sustaining any penalties in terms of priming volume and blood–foreign surface interaction. Figure 4 visualizes this comparison, showcasing a 0.7 m^2^ silicon-coated module which, under hyperbaric gas supply, yields an oxygen uptake capability equivalent to that of its sibling, with twice the surface area and operation at normobaric conditions.

**Figure 5:**
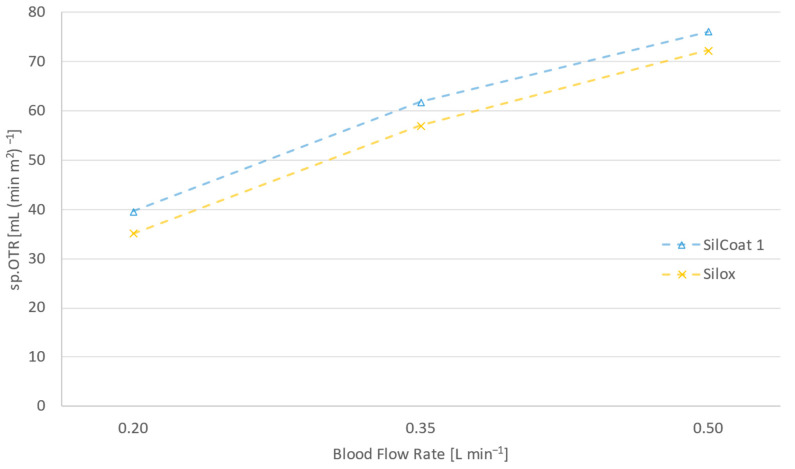
Surface area specific oxygen transfer rates of a silicon-coated module and a solid silicone capillary oxygenator at 0.2, 0.35 and 0.5 L min^−1^ blood flow rate and hyperbaric operation.

Figure 5 draws attention to the significance of silicone thickness in gas diffusivity. In the case of SilCoat modules, the silicone layer is on average 10 times thinner than the capillary wall of the Silox membrane.

**Figure 6:**
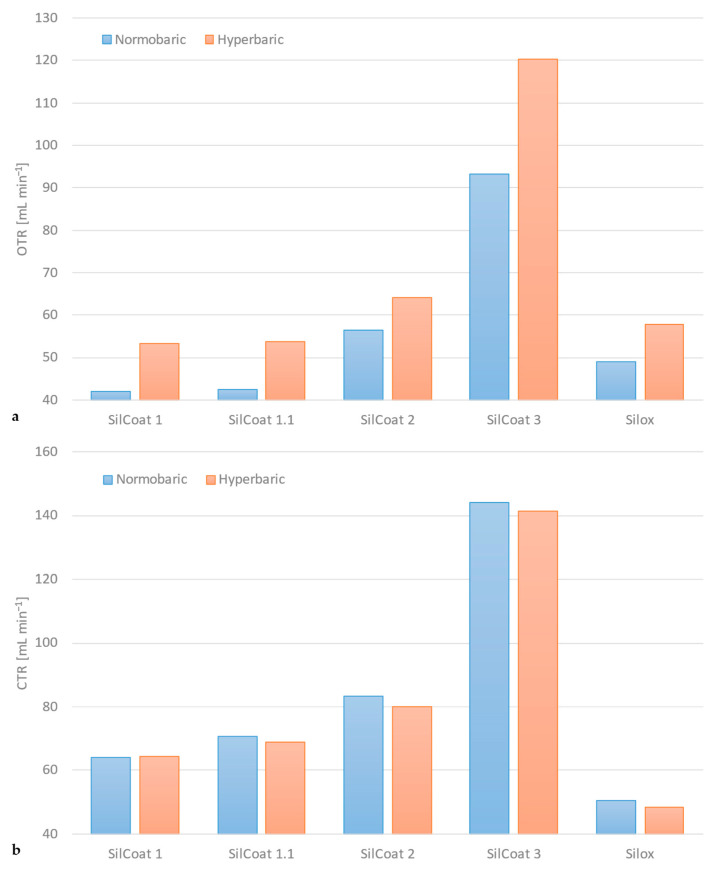
Overview of the gas exchange performance of every device tested in hyperbaric and normobaric conditions (SilCoat 3 at Q_B_ = 1.0 L min^−1^, Q_B_ = 0.5 L min^−1^ for all other modules, Q_G_/Q_B_ ≥ 5:1): (**a**) oxygen transfer rate, (**b**) carbon dioxide transfer rate.

Figure 6a portrays a synopsis of the enhanced gas exchange performance that the principle of hyperbaric gas delivery presents itself with, contrary to conventional normobaric sweep gas supply. This instance depicts the oxygen transfer rate of all tested devices under normo- versus hyperbaric gas delivery at the maximum blood flow rate (variable depending on module size).

As previously mentioned, the carbon dioxide transfer rate deteriorates somewhat during hyperbaric operation. Yet, application of higher gas flow/blood flow ratios mitigates this handicap, as Figure 6b denotes. The difference in carbon dioxide elimination rate across the range of tested modules is less than 3% between normo- and hyperbaric operation.

Finally, no gas bubbles were detected in arterial blood throughout this study, corroborating the safety of the experimental protocol and ratifying once more the reliability of silicone-coated fibers for gas exchange applications.

## 4. Discussion

The advent of capillary membranes and the development of new technologies for the mass production of hollow fiber mats unquestionably reshaped the face of extracorporeal gas exchange in medical applications [13]. Yet, like a landslide, this revolution squashed any other products and/or ideas, eventually bringing about a stalemate, both in research and in product development. The most significant contributions of the past decade concentrate on improving the perfusion of the fiber bundle, either through augmentation of secondary flow, or simply by thwarting stagnation zones via design optimization of the flow path and the blood chamber [14,15,16,17]. Albeit innovative, these concepts offer an ameliorated gas exchange performance (often in tandem with hemocompatibility) for a very specific fiber bundle design, which limits their appositeness. Moreover, any perfusion-oriented amendment suffers the penalty of a predetermined cap in maximal gas exchange performance imposed by the laws of nature, the mass transfer coefficient can only be refined to a certain degree.

On these grounds, this study shifted its attention towards the most neglected parameter of mass transfer, namely, the driving force itself. Much as a gas mixture consisting of pure oxygen (*FiO*_2_ = 1.0) is incontestably more efficacious than air (*FiO*_2_ = 0.21), so is hyperbaric gas delivery superior to normobaric methods. Certainly, hyperbaric applications in extracorporeal gas exchange necessitate purely diffusive membranes to operate safely, but as it turns out, this is not at all a high price to pay.

Despite sounding counterintuitive in terms of gas transfer efficacy, solid membranes have in fact facilitated the conduct long-term extracorporeal gas exchange therapies by notably reducing the risk of plasma leakage. Hence, contemplating PMP as a potential candidate for controlled hyperbaric operation within the scope of this study would have been a perfectly valid argument. However, the thread knots holding the PMP capillaries together at fixed intervals often damage the merely 1 μm thin solid skin, and microcracks start to develop [18]. Furthermore, despite its widespread applicability nowadays, PMP does not in fact prevail in terms of selectivity, as Table 3 highlights. In light of the above, it is safe to claim that the existing capillary membrane technology does not put forward any truly durable, efficient, and affordable products for hyperbaric applications.

On the contrary, solid-silicone and silicone-treated gas exchangers are ideally suited for both prolonged applications and hyperbaric gas delivery regimens. Silicone-coated modules in particular, featuring a silicone layer of less than 20 μm, guarantee unimpeded diffusion rates for both oxygen and carbon dioxide, on top of truly leakage-free operation [9,10]. The findings of the hyperbaric gas supply study communicated here once again underline the reliability and applicability of silicone-coated microporous fibers.

Raising the pressure in the gas compartment by 300 mmHg gives rise to an almost 40% increase in oxygen concentration. In other words, instead of having a partial pressure of 760 mmHg, the hyperbaric gas phase has a *pO*_2_ exceeding the 1000 mmHg threshold.

Figure 3a states that a gas exchanger designed for blood flow rates of up to 1 L min^−1^, with an acceptable shear rate, emphatically fails to deliver adequate gas transfer above 0.5 L min^−1^ (*sO*_2_ = 89% and 81% at 0.75 and 1.0 L min^−1^, respectively), falling short of the OTR max reference. Application of pressurized gas supply instantly transforms this module, amplifying its gas exchange capacity by up to 129% and expanding its application range way past the normobaric range (*sO*_2_ = 97% at 1.0 L min^−1^).

Figure 3b explicates the performance upsurge by juxtaposing oxygen saturation and the partial pressure of arterial blood under normo- and hyperbaric operation. Evidently, the partial pressure of oxygen receives a nearly 6-fold boost under a hyperbaric aeration regime, whereas saturation remains high even at 50 and 100% above nominal blood flow rate (500 mL min^−1^).

In fact, the magnitude of oxygen transfer rate (OTR) improvement is such that it actually permits a surface area reduction—even halving—as Figure 4 indicates. This translates to strikingly lower priming volumes and reduced blood contact with foreign surfaces. In turn, these effects ought to dampen the occurrence of hemolysis.

Notwithstanding the remarkably thin silicone layer of the silicone-coated modules, Figure 5 discloses a meager 5–10% profit in gas transfer over the solid-silicone capillaries of Silox. This slight improvement is attributed to the cell-free marginal layer, which causes a distinctly higher resistance to gas diffusion than membrane thickness itself.

The maximum performance gain from hyperbaric gas delivery across the range of tested modules varies between 10 and 29%, as Figure 6a suggests. The notable divergence and the extrema can be attributed to the test protocol itself. Testing some of the gas exchangers at proportionally higher blood flow rates or using a positive pressure higher than 300 mmHg (400 mbar) would most likely magnify both the minimum and maximum performance, and presumably dampen the variance. The former has been avoided in pursuance of remaining as close to the nominal blood flow rate values and subsequently within the physiological shear rate range of each apparatus. The latter has been intentionally restricted, since a 40% rise in sweep gas pressure might already be alarming enough for clinicians and perfusionists alike.

Hyperbaric sweep gas delivery is not a panacea for extracorporeal gas exchange since it hinders the migration of carbon dioxide from blood into the gas phase. Yet, sufficiently high gas flow/blood flow ratios—akin to physiological hyperventilation—accomplish the task of adequate CO_2_ elimination. As Figure 6b delineates, the tested gas exchangers are highly efficient in eliminating CO_2_ in every instance, although normobaric operation favors CO_2_ removal ever so slightly.

**Figure 7:**
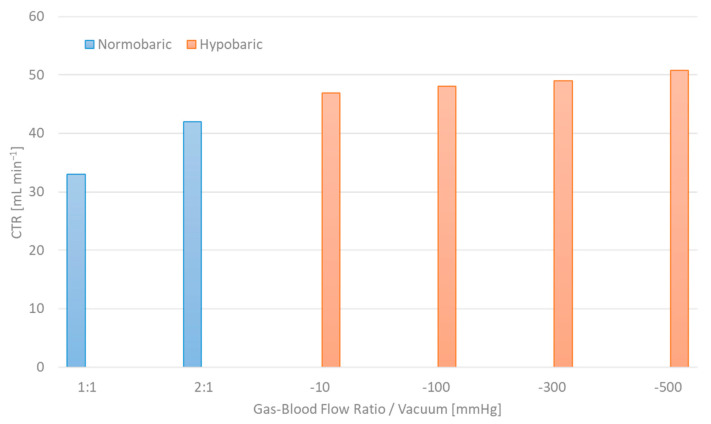
Carbon dioxide elimination efficacy of hypobaric operation in contrast to conventional “hyperventilation” approach.

Further decarboxylation can be achieved simply by inverting the principle of hyperbaric gas supply; namely, by applying vacuum at the oxygenator’s gas outlet, while constricting the gas inlet. The ensuing negative pressure in the gas chamber promotes the CO_2_ transfer rate by artificially amplifying the diffusion potential [21]. Figure 7 encapsulates the carbon dioxide removal potential of the Silox module at 1:1 and 2:1 gas flow/blood flow ratios in contrast to hypobaric operation at −10, −100, −300 and −500 mmHg, thus providing substantial proof of the concept’s feasibility. Given that CO_2_ elimination plateaus for gas flow/blood flow ratios above 15:1, the combined application of vacuum and “hyperventilation” could offer optimum carbon dioxide transfer rates.

Naturally, hypobaric operation is counterintuitive in terms of oxygenation. However, a synergistic approach, where different modules or otherwise distinct gas compartments are individually treated with either hyperbaric or hypobaric gas supply, can potentially lead to peak gas exchange performance. Further development and optimization of the gas flow constriction system ought to refine the adjustability of hyper- and hypobaric aeration regimes, laying the groundwork for full-scale in vitro and in vivo studies.

## 5. Conclusions

This study introduces an innovative approach to extracorporeal gas exchange, promising radically high levels of performance. Constriction of an oxygenator’s gas outlet leads to pressurization of the gas chamber, enhancing the concentration gradient of oxygen between the two fluids flowing on each side of the membrane. It is imperative that the membrane is of a purely diffusive nature in order to avoid gas leakage, which might induce the formation of gas bubbles in bloodstream. Silicone, an acknowledged bio- and hemocompatible material [22,23,24,25] frequently implemented in extracorporeal gas exchange [22,24,25,26] as a result of its inherent high gas permeability [18,27,28], presents itself as the ideal candidate for hyperbaric aeration applications. The in vitro investigations brought forth irrefutable evidence of the concept’s merit, confirming the assumption of ample gas-exchange performance gains. The fact that the principle offers surface area downsizing as a side benefit exponentially increases the value added. Ultimately, the possibility of combining hyperbaric gas supply with “hyperventilation”, or with hypobaric aeration in modular configurations, promises soaring performance, whilst simultaneously expanding the applicability of the concept.

## 6. Patents

Patents were filed on behalf of RWTH Aachen University.

## Figures and Tables

**Table 1:** Technical characteristics of the tested gas exchangers.

Model	n	Surface Area A_eff_ [m^2^]	Priming Volume V_prim_ [mL]	Fiber Length L_eff_ [mm]	Fiber Size d_in_/t_w_ [μm]	N_fiber_	Blood Flow Rate Q_B_ [L min^−1^]
Silox	3	0.8	50	200	200/100	9000	0.2–0.5
SilCoat 1	3	0.7	42	225	200/40	4951	0.2–0.5
SilCoat 1.1	3	0.7	52	225	220/35	4501	0.2–0.5
SilCoat 2	3	1.4	100	225	220/35	9002	0.2–0.5
SilCoat 3	3	1.8	130	225	220/35	11,574	0.5–1.0

**Table 2:** Venous blood–gas data as per adapted experimental protocol.

*pCO*_2_ [mmHg]	*sO*_2_ [%]	Base Excess [mmol dL^−1^]	ϑ [°C]	Hb [g dL^−1^]	Q_B_ [mL/min]	Q_G_/Q_B_	P_G_ [mmHg]
48 ± 2	30 ± 2	0 ± 2	37 ± 1	12.8 ± 0.2	200–1000	≥5:1	atm./+300

**Table 3:** Selectivity of commonly implemented materials in gas exchange [19,20].

Gas/Material	Permeability Coefficient 3.35×10−16mol·mm2·s·Pa		
	PP	PMP	Silicone
Oxygen	2.2	32.3	605
Carbon Dioxide	9.2	92.6	3240

## Data Availability

The raw data supporting the conclusions of this article will be made available by the authors, without undue reservation.
